# Consent for Brain Tissue Donation after Intracerebral Haemorrhage: A Community-Based Study

**DOI:** 10.1371/journal.pone.0135043

**Published:** 2015-08-24

**Authors:** Neshika Samarasekera, Christine Lerpiniere, Arthur F. Fonville, Andrew J. Farrall, Joanna M. Wardlaw, Philip M. White, Antonia Torgersen, James W. Ironside, Colin Smith, Rustam Al-Shahi Salman, Lothian Audit of the Treatment of Cerebral Haemorrhage (LATCH) collaborators

**Affiliations:** 1 Division of Clinical Neurosciences, Centre for Clinical Brain Sciences, University of Edinburgh, Edinburgh, United Kingdom; 2 Brain Research Imaging Centre, University of Edinburgh, Edinburgh, United Kingdom; 3 Centre for Cognitive Ageing and Cognitive Epidemiology, University of Edinburgh, Edinburgh, United Kingdom; Cambridge Institute for Medical Research, UNITED KINGDOM

## Abstract

**Background:**

Spontaneous intracerebral haemorrhage is a devastating form of stroke and its incidence increases with age. Obtaining brain tissue following intracerebral haemorrhage helps to understand its cause. Given declining autopsy rates worldwide, the feasibility of establishing an autopsy-based collection and its generalisability are uncertain.

**Methods:**

We used multiple overlapping sources of case ascertainment to identify every adult diagnosed with intracerebral haemorrhage between 1^st^ June 2010-31^st^ May 2012, whilst resident in the Lothian region of Scotland. We sought consent from patients with intracerebral haemorrhage (or their nearest relative if the patient lacked mental capacity) to conduct a research autopsy.

**Results:**

Of 295 adults with acute intracerebral haemorrhage, 110 (37%) could not be approached to consider donation. Of 185 adults/relatives approached, 91 (49%) consented to research autopsy. There were no differences in baseline demographic variables or markers of intracerebral haemorrhage severity between consenters and non-consenters. Adults who died and became donors (n = 46) differed from the rest of the cohort (n = 249) by being older (median age 80, IQR 76–86 vs. 75, IQR 65–83, p = 0.002) and having larger haemorrhages (median volume 23ml, IQR 13–50 vs. 13ml, IQR 4–40; p = 0.002).

**Conclusions:**

Nearly half of those approached consent to brain tissue donation after acute intracerebral haemorrhage. The characteristics of adults who gave consent were comparable to those in an entire community, although those who donate early are older and have larger haemorrhage volumes.

## Introduction

Spontaneous intracerebral haemorrhage (ICH) is a devastating form of stroke which carries a one month case fatality of approximately 42%[1] and has an increasing incidence with age. Given the ageing population in the UK and other developed countries,[2] ICH incidence is likely to increase.

Although investigations may help to identify a specific ICH cause such as a tumour (secondary ICH), ~80% ICHs do not have an apparent cause (so called ‘primary ICH’) and are attributed to small vessel vasculopathies on the basis of a patient’s clinical risk factors and radiological features of the ICH.[3] Examination of brain tissue may help us to understand more about ICH cause, in particular the contribution of small vessel vasculopathies, which can still only be diagnosed with certainty using pathological specimens.

Research using brain tissue samples has been hampered by declining post-mortem examination rates,[4] various organ retention scandals and an incomplete understanding of factors which may influence consent.[5] Studies of organ donation for transplant have identified the timing of the request and whether the request is made ‘collaboratively’ by both the organ donation and clinical teams as being predictors of obtaining consent.[6] A recent systematic review[5] identified eleven studies of brain donation for research but no previous studies in adults with stroke. The heterogeneity of previous studies both in terms of their donor populations and mode of seeking consent precludes firm conclusions but demographic factors such as donor age, sex, marital status, level of education and social class do not seem to influence consent.

In a community-based study of brain tissue donation from adults dying after ICH, we sought to ascertain: a) the proportion giving consent, b) whether any characteristics of participants, their ICH or the consent process were associated with giving consent and c) whether adults approached to give consent, those who consented and those who eventually became brain donors were representative of the entire cohort of patients with ICH.

## Methods

### Community-based inception cohort study of ICH

The Lothian Audit of the Treatment of Cerebral Hemorrhage (LATCH) ascertained all residents in the Lothian Health board region of Scotland (mid-2010 population aged ≥16 years was 695,335) who were aged ≥16 years at the time they were diagnosed with first-ever or recurrent ICH confirmed by brain imaging or pathology between 1st June 2010 and 31st May 2012 inclusive. We excluded adults with exclusively extra-axial intracranial haemorrhage or ICH definitely attributable to trauma or hemorrhagic transformation of an ischaemic stroke.[7]

We identified incident ICH cases using multiple overlapping sources of case ascertainment.[7] The NHS Lothian Caldicott Guardian approved LATCH. Patients in NHS Lothian were informed about the use of their data for audit, and information leaflets about LATCH were distributed to inform patients and their carers about their right to opt out. Analyses of an anonymised dataset, extracted from the audit database held on NHS and University servers, did not require research ethics committee approval.

### Consent for brain tissue donation

Patients with ICH ascertained by LATCH had the opportunity to consent to participate in the Lothian IntraCerebral Haemorrhage, Pathology, Imaging and Neurological Outcome (LINCHPIN) study, a prospective community-based research study examining the causes of ICH using research autopsy in case of death. If the patient lacked mental capacity as defined by the Adults with Incapacity (Scotland) Act 2000, we sought consent for brain tissue donation from their nearest relative or legal representative in accordance with the statutory requirements of the Human Tissue (Scotland) Act 2006 which requires consent to be sought before the use of a deceased person’s organs, tissues or cells for medical research.

We sought consent in person, tailoring information to the participant’s wishes. We provided participants or their relatives with an information leaflet detailing the autopsy process and discussed both generic consent issues (such as the voluntary nature of participation and confidentiality) and information regarding the process of brain donation on many occasions if required to ensure that all concerns were addressed.[5] We sought written informed consent from the donor or their next-of-kin for one centimetre cubed brain tissue samples to be taken from all lobes of the brain, the cerebellum and brainstem to be used for research purposes, with the remainder of the brain returned to the donor’s body. The Scotland A Research Ethics Committee approved the study (10/MRE00/23).

### Clinical information

We collected clinical variables by interviewing patients or their families at the time of presentation and reviewing primary care and hospital records. We classified ethnicity as either white or non-white and level of education as either basic (school only) or further/higher (college, apprenticeships or university).

We assessed socioeconomic status using the Scottish Index of Multiple Deprivation (http://www.scotland.gov.uk/Topics/Statistics/SIMD/BackgroundMethodology; 2012) which ranks regions by postcode from the most deprived (rank one) to the least deprived (rank 20) using a combination of 38 indicators across the following domains: income, employment, health, education, skills and training, housing, geographic access and crime.

We defined ICH location as either ‘lobar’ or ‘non-lobar’. At least one experienced consultant neuroradiologist reviewed diagnostic brain imaging and classified ICH location as ‘non-lobar’ if an adult had a single infratentorial ICH (located in the brainstem or cerebellum), a single supratentorial deep ICH (located in the basal ganglia, internal or external capsule or thalamus without extension to a lobar area), or multiple ICHs in solely non-lobar locations (either supratentorial deep or infratentorial). All other ICHs were ‘lobar’.

### Statistical analysis

Based upon previous studies of consent for brain tissue donation,[5] we compared demographic, clinical and radiological characteristics in adults who a) were approached to consider brain donation vs. those who were not, b) in those who consented to brain donation vs. those declined, c) in those who consented vs. the remainder of the cohort and d) in donors vs. the remainder of the cohort. We used parametric statistics when characteristics conformed to a normal distribution and non-parametric statistics when they did not.

For adults who underwent brain imaging (and were not diagnosed at autopsy) we calculated ICH volume using the first CT brain scan after the adult’s symptom onset using the ABC/2 method.[8] We documented contemporaneously the reason(s) cited by participants or their nearest relative for giving or declining brain donation.

From existing knowledge from studies of organ donation for transplantation on features of the consent process which may influence whether consent is obtained,[6] we selected *a priori* three variables to compare between those who consented and those who declined: whether a member of the clinical team or the research team first discussed post-mortem examination, the interval between the date of the ICH and the date brain donation was first discussed, and whether consent was sought from the patient or their nearest relative.

## Results

**Fig 1:**
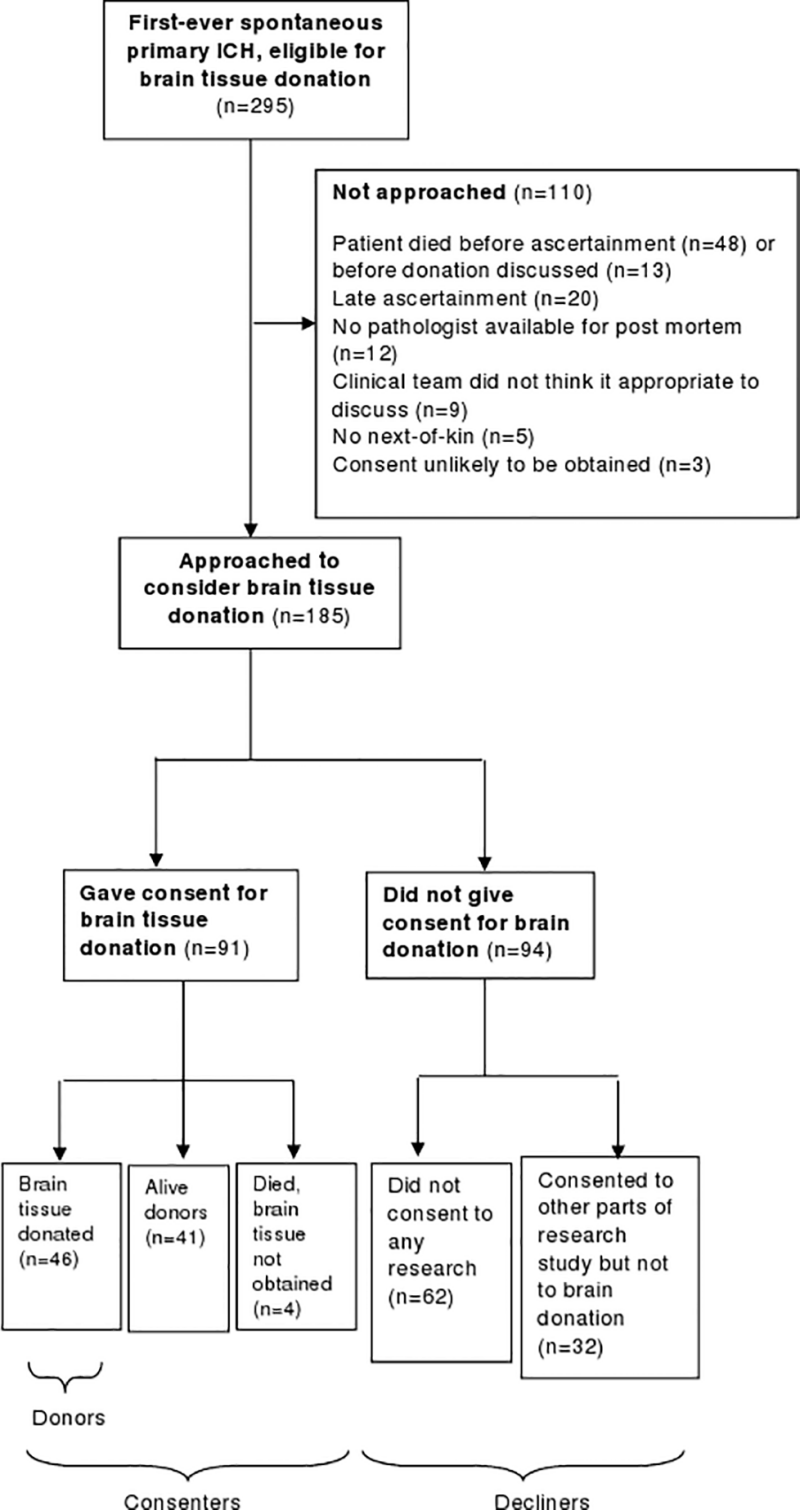
Flowchart of adults with spontaneous ICH eligible to donate brain tissue from 1^st^ June 2010-31^st^ May 2012.

Of 295 adults with spontaneous primary ICH (who were eligible for brain donation) from 1^st^ June 2010-31^st^ May 2012, 110 (37%) were not approached, leaving 185 adults of whom 91 (49%; 95% CI 42–56%)) consented to brain tissue donation in case of death (Fig 1). The most frequent reason that participants were not approached was that they had died before being ascertained or before brain tissue donation was discussed (n = 61; 55%). 20 adults (18%) were ascertained late (at least two months after the date of their ICH) when we no longer considered it appropriate to approach participants to request brain donation.

### Approached vs. not approached to consider brain donation

Adults who were not approached had a lower socioeconomic status but other baseline demographic characteristics were similar in both groups (Table 1). Adults not approached had significantly lower conscious levels on admission with larger ICHs which were more likely to rupture into the ventricles.

**Table 1:** Baseline characteristics of adults with spontaneous ICH approached and not approached to consider brain donation.

	Approached to consider brain donation (n = 185)	Not approached to consider brain donation (n = 110)	p value
**Sex** male, (%)	90 (49)	51 (46)	0.70
**Age** (years); median (IQR)	77 (68–82)	77 (65–85)	0.32
**Ethnicity** non-white, (%)	7 (4)	1 (1)	0.27
**Socio-economic status** * (postcode rank)	13 (7–18)	10 (6–16)	0.03
**Admission GCS score** **; median (IQR)	14 (12–15)	10 (4–15)	<0.001
**Mean arterial pressure** *** (mmHg) mean (SD)	115 (25)	119 (29)	0.22
**Lobar ICH location**	99 (64)	56 (51)	0.67
**Intraventricular extension** ^	74 (40)	67 (61)	<0.001
**ICH volume** ^ (ml); median (IQR)	14 (5–30)	21 (5–58)	0.04

*Scottish index of multiple deprivation categories (2012); higher postcode rank indicates lower deprivation index; missing in two cases

**Glasgow Coma Scale score—missing in nine cases; four of whom were not admitted to hospital

***missing in 22 cases

^not applicable in five cases in which the diagnosis was confirmed at post-mortem examination

### Consented to brain donation vs. declined consent

Baseline demographic and clinical variables and features of the consent process were similar in those who consented to brain donation vs. those who declined (Table 2).

**Table 2:** Demographic and clinical variables and characteristics of the consent process in adults who give consent to brain tissue donation and those who decline donation.

	Consented to brain donation (n = 91)	Declined brain donation (n = 94)	p value
**Non-modifiable patient characteristics**			
**Sex** male, (%)	48 (53)	42 (45)	0.27
**Age** (years); median (IQR)	76 (69–84)	77 (67–82)	0.33
**Ethnicity** non-white, (%)	1 (1)	6 (6)	0.12
**Socio-economic status** *	14 (7–18)	12 (7–17)	0.18
**Donor education** **; further or higher, (%)	24 (26)	21 (22)	0.50
**Non-modifiable ICH characteristics**			
**Admission Glasgow Coma Scale score**; median (IQR)	14 (11–15)	14 (13–15)	0.04
**Intraventricular extension** *** Yes (n, %)	39 (43)	35 (37)	0.40
**ICH volume** ***(ml); median (IQR)	15 (5–30)	12 (5–30)	0.28
**Modifiable characteristics of the consent process**			
**Approached by clinical team and researcher** (vs. researcher alone)	8 (9)	4 (4)	0.21
**Interval between date of ICH and date post-mortem examination discussed** ^ (days; median (IQR))	4 (2–12)	5 (2–14)	0.99
**Approached nearest relative** (vs. participant)	61 (67)	60 (55)	0.65

*missing in two cases

** missing in 22 cases

***not applicable in one case of ICH which was diagnosed at post-mortem examination

^missing in four cases

### Consented to brain donation vs. the remainder of the cohort

Adults who consented had a higher socioeconomic status in comparison to the rest of the cohort but there were no other differences in either demographic or clinical characteristics (Table 3).

**Table 3:** Demographic and clinical characteristics in those who consented to brain donation vs. remainder of the cohort.

	Consented to brain donation (n = 91)	Did not consent to brain donation (n = 204)	p value
**Sex** male, (%)	48 (53)	93 (46)	0.25
**Age** (years); median (IQR)	76 (69–84)	77 (67–83)	0.78
**Ethnicity** non-white, (%)	1 (1)	7 (3)	0.45
**Socio-economic status** (postcode)*	14 (7–18)	11 (6–16)	0.03
**Admission GCS score** ** median (IQR)	14 (11–15)	14 (8–15)	0.10
**Mean arterial pressure** *** (mmHg) mean (SD)	116 (23)	116 (28)	0.97
**Lobar ICH location**	54 (59)	101 (50)	0.12
**Intraventricular extension** ^	39 (43)	102 (50)	0.23
**ICH volume** ^ (ml);median (IQR)	15 (5–30)	15 (5–42)	0.84

*missing in two cases

**Glasgow Coma Scale score—missing in nine cases; four of whom were not admitted to hospital

***missing in 22 cases

^not applicable in five cases in which the diagnosis was confirmed at post-mortem examination

### Reasons cited for giving and declining consent for brain donation

Participants or their nearest relatives cited a median number of one theme (IQR 1–1) both when consenting to, and declining, brain donation (Tables 4 and 5). The most frequent reason given was the potential benefit as a result of their donation to others with the same condition (Table 4). The most frequent reason given for declining to participate was that the person asked did not feel able to make a decision (Table 5).

**Table 4:** Reasons given for consent to brain donation among 91 consenters.

Reasons given for donation (most common first)	n (%)
Potential benefit to others with the same condition	52 (57)
Wished to participate in a research study	24 (26)
Perception of body as merely a physical shell	10 (11)
Offer an explanation for the intracerebral haemorrhage	5 (5)
Consistent with prior wish to donate body to medical science	5 (5)
Wish to repay medical care provided	4 (4)
No objection to research post-mortem examination	2 (2)
Potential benefit to patient from participation in a research study	2 (2)
No reason given	2 (2)

**Table 5:** Reasons given for declining brain donation among 94 decliners.

Reasons given for refusal to consent	n (%)
Nearest relative unable to decide	17 (18)
Did not know what the potential donor’s wishes would be	7 (8)
too upset by diagnosis of intracerebral haemorrhage	5 (5)
No reason given	5 (5)
Did not wish to be involved in a research study	13 (14)
Brain donation is ‘too invasive’ or ‘not something they wished to put their next-of-kin through’	13 (14)
Consent was incompatible with previously expressed wishes	9 (10)
Conflict in family regarding post-mortem examination decision	9 (10)
Patient’s deteriorating condition or other comorbidities	6 (6)
Consent was incompatible with spiritual or religious beliefs	5 (5)
Dislike the idea of brain tissue donation	4 (4)
Autopsy not considered because thought to be synonymous with death	2 (2)
Concerns regarding autopsy procedure or disfigurement	2 (2)
Dissatisfaction with medical care	1 (1)
Dissatisfaction with previous discussion regarding organ donation	1 (1)
No reason given	15 (16)

### Donors vs. the rest of the cohort

Donors were older and had larger ICHs in comparison to the remainder of the cohort (Table 6). 14 donors survived for longer than three months after their ICH. In a sensitivity analysis restricted to donors who died within three months of their ICH the results were unchanged.

**Table 6:** Demographic and clinical variables in donors and non-donors.

	Donors (n = 46)	Non-donors (n = 249)	p value
**Sex** (male), (%)	25 (54)	116 (47)	0.33
**Age** (years); median (IQR)	80 (76–86)	75 (65–83)	0.002
**Ethnicity** (non-white); (%)	0 (0)	8 (3)	0.62
**Socio-economic status** * (postcode rank)	13 (6–18)	12 (6–17)	0.45
**GCS on admission** ** median (IQR)	13 (10–14)	14 (9–15)	0.14
**Intraventricular extension** ***; n (%)	25 (56)	116 (47)	0.31
**ICH volume** *** median (IQR)	23 (13–50)	13 (4–40)	0.002

*missing in two cases

** Glasgow Coma Scale score—missing in nine cases, four of whom were not admitted to hospital

***missing in five cases which were diagnosed at post-mortem examination

## Discussion

### Main findings

In a prospective, community-based inception cohort study, 49% of adults with ICH or their relatives who were approached, later consented to donate brain tissue at a research autopsy. Adults who were approached had a higher socioeconomic status and less severe ICHs than those not approached. There were no significant differences between those who consented and those who declined brain donation. Those who consented had a higher socioeconomic status in comparison to the remainder of the cohort. Donors were older and had larger ICHs in comparison to the remainder of the cohort.

### Strengths of the study

To the best of our knowledge, this is the first study to report the proportions consenting to brain donation for research purposes in a population-based cohort of participants with any form of stroke.

The population-based design with comprehensive case ascertainment permits an evaluation of the representativeness of those who were approached to consider brain donation, those who consented and those who eventually became brain donors in comparison to the remainder of the cohort. Participants were well phenotyped since the clinical history was ascertained prospectively prior to the outcome (obtaining consent) and ICH-related variables were ascertained blind to the outcome. There were few differences between those who consented, donors and the rest of the cohort and differences were predominantly attributable to disease severity. These may disappear when those who consented die after milder ICHs.

### Weaknesses of the study

The study took place in a predominantly white population in South East Scotland so we could not examine the role of ethnicity and differing cultural beliefs on donation. Although participants may be more likely to consent to retention of tissue samples rather than their whole brain,[9] we were unable to explore this in our cohort. ICH has a high early case fatality so we were unable to approach ~ one third of adults who had more extensive ICHs in comparison to those approached, the majority of whom had died either before ascertainment or before brain tissue donation could be discussed. We did not explore demographic or cultural factors pertaining to nearest relatives although these may influence participants’ perceptions of brain donation. In addition, the study was largely quantitative in nature and studying the influence of cultural beliefs and personal experience on donation is likely to be more suited to qualitative methodologies.

### Comparison with other studies

The proportion of adults consenting to brain tissue donation in our study is comparable to other studies of brain tissue donation which have taken place predominantly in the setting of chronic diseases and healthy populations. Although two studies have reported much higher consent proportions,[9,10] one of these studies predated the organ retention scandals of the mid-1990’s (that may have contributed to a subsequent decline in post-mortem examinations)[10] and the second study was conducted in the setting of sudden death[9] when a legal post-mortem examination is mandated to establish the cause of death.

In our study, there was no difference in demographic variables between those who consented and those who declined (apart consenters having a higher socioeconomic status, which may relate to the characteristics of the catchment population of the hospital at which the research team was based); other studies of brain donation have not shown any influence of donor sex, age, level of education or socioeconomic status on the decision to consent.[5] There was no difference between those who consented and those who declined brain donation regarding the person making the approach or the timing of it. Since a joint approach by the clinical and research teams was rare in this cohort, it may be that there was insufficient power to demonstrate a difference. One study of sudden death reported that the longer the interval between death and the approach for brain donation, the more likely that consent would be given[11] but we did not find this in our cohort.

As in our cohort, prospective donors have commonly reported altruistic motivations including a desire to help others[10–15] or support research [9,13,15–17] in their decision to donate. The most frequently cited reason for not giving consent in our cohort was that donors or their nearest relatives felt unable to make a decision, which was related in part to the distress caused by a diagnosis of ICH and a lack of clarity regarding what the donor’s wishes would have been. This has not been cited in other studies of brain donation which have frequently been conducted in either healthy participants or those with chronic neurological diseases when the decision to donate does not usually have to be made rapidly or unexpectedly.

### Meaning of the study

An ongoing dilemma is how to increase the number of brains donated for research, a priority acknowledged by BrainNet Europe and the UK Medical Research Council.[18] This study demonstrates the feasibility of seeking consent for brain donation in acute neurological diseases like ICH and forming a reasonably generalisable brain bank.

Although reasons for refusing consent which were likely irreversible predominated in our cohort, a modifiable reason was not knowing what the participant’s wishes would have been. It is essential to increase public awareness of organ donation for for both research and transplantation with the aim of empowering people to reach their own decisions and communicate this to relatives when they still have the mental capacity to do so.[19]

The lack of association between demographic characteristics and consent in this and other studies[5] suggests that deciding whether to consent may be related to personal beliefs and unquantifiable aspects of the consent process. Researchers or clinicians should avoid presuming on the basis of demographic variables that either consent will not be obtained or asking will cause upset. [18,20]

### Future directions

We will re-assess the generalisability of this brain bank when more consenters die long after milder ICHs. Future studies should examine brain donation in different populations for other neurological diseases. Qualitative studies should explore the influence of personal beliefs and experiences on consent, including factors which are difficult to measure quantitatively such as the expertise and interpersonal skills of the person seeking consent and the donor’s perceived satisfaction with clinical care.[6] Nearest relatives’ experiences of the consent and donation process could help assess whether their expectations were met, whether they had any regrets regarding donation and any improvements that could be made. Ultimately, this will be essential to ensure that brain banks are adequately representative of the neurological diseases that are set to become more common in an ageing population.
